# Conjugation of Amisulpride, an Anti-Psychotic Agent, with 5-Aminosalicylic Acid via an Azo Bond Yields an Orally Active Mutual Prodrug against Rat Colitis

**DOI:** 10.3390/pharmaceutics11110585

**Published:** 2019-11-07

**Authors:** Wooseong Kim, Dayoon Kim, Seongkeun Jeong, Sanghyun Ju, Hanju Lee, Soojin Kim, Jin-Wook Yoo, In-Soo Yoon, Yunjin Jung

**Affiliations:** College of Pharmacy, Pusan National University, Busan 46241, Korea; b04420@nate.com (W.K.); ekdbs27@naver.com (D.K.); aofhd86@naver.com (S.K.J.); jsh141002@naver.com (S.H.J.); purity4127@naver.com (H.L.); wldhel3507@hanmail.net (S.K.); jinwook@pusan.ac.kr (J.-W.Y.); insoo.yoon@pusan.ac.kr (I.-S.Y.)

**Keywords:** amisulpride, 5-Aminosalicylic acid, mutual prodru, colon-specific drug delivery, inflammatory bowel disease

## Abstract

Amisulpride (ASP), an anti-psychotic agent, is a pharmacologically equivalent to sulpiride (SP). Because SP demonstrates anti-ulcer and anti-colitic activities, ASP with an aniline moiety was azo-coupled to salicylic acid to generate 5-(aminoethanoylsulfamoyl)-*N*-[(1-ethylpyrrolidin-2-yl)methyl]-2-methoxybenzamide (ASP-azo-ASA), with the expectation that it would act as a colon-specific mutual prodrug against colitis. Following a 24 h incubation, approximately 80% of ASP-azo-ASA was cleaved to form ASP and 5-aminosalicylic acid (5-ASA) in the cecal contents, whereas it remained stable in the small intestinal contents. Oral gavage of ASP-azo-ASA (oral ASP-azo-ASA) delivered 5-ASA to the cecum to levels comparable with those observed for sulfasalazine (SSZ; clinical colon-specific prodrug of 5-ASA) and without detectable concentrations of ASP in the blood, indicating efficient colonic delivery. Oral ASP-azo-ASA ameliorated 2, 4-dinitrobenzenesulfonic acid hydrate (DNBS)-induced colitis in rats more effectively than oral SSZ. Additionally, oral ASP-azo-ASA lowered the levels of inflammatory mediators in the inflamed distal colon more effectively than oral SSZ. Combined treatment with 5-ASA and ASP via the rectal route more effectively reversed colonic damage and inflammation than treatment with 5-ASA or ASP alone, confirming the mutual anti-colitic actions of 5-ASA and ASP. In conclusion, ASP-azo-ASA is an orally active mutual prodrug against rat colitis with limited systemic absorption of ASP.

## 1. Introduction

Inflammatory bowel disease (IBD) is an idiopathic and chronic inflammatory disorder of the gastrointestinal (GI) tract. Ulcerative colitis (UC) and Crohn’s disease (CD) are the principal types of IBD. Along with differences in the pathologic features of UC and CD, inflammatory lesions caused by these diseases are differentially distributed in the GI tract. UC primarily involves the colon and rectum, while CD affects any part of the GI tract, from the mouth to the anus [1]. Although the exact molecular etiology remains to be elucidated, IBD is thought to be caused by the interaction of environmental and genetic factors, leading to excessive immune responses to host intestinal microflora and resulting in destructive inflammation [2,3]. 

5-Aminosalicylic acid (5-ASA, mesalazine) is an anti-inflammatory drug used for the induction and maintenance of remission in patients with mild-to-moderate IBD. Although 5-ASA is still the mainstay for IBD drug therapy, its low anti-colitic efficacy limits its therapeutic usefulness, and there is often a need to switch or combine medication with other anti-IBD drugs, such as immunosuppressants or glucocorticoids, which can elicit serious side effects [4,5]. In addition, 5-ASA is not suitable for oral use because of the risk of side effects associated with the extensive systemic absorption of 5-ASA. Therefore, 5-ASA should be pharmaceutically and chemically formulated for target site (large intestine)-specific delivery [5,6]. However, compared with other anti-IBD drugs, including biologics, 5-ASA is attractive in aspects of cost and safety [4]. Developing a 5-ASA-based drug with improved anti-colitic efficacy may provide a further therapeutic option, which is patient-friendly, for the treatment of moderate-to-severe IBD. 

Colon-specific drug delivery tends to increase the colonic concentration of the drug and reduce its systemic absorption, resulting in increase in therapeutic availability at the target site and reduced risk of systemic side effects [7,8]. Therefore, colon-specific drug delivery is applied to development of drugs for the treatment of colonic diseases, such as UC and CD. Chemical (prodrug) and pharmaceutical approaches are utilized to grant colon specificity to drugs [7]. Sulfasalazine (SSZ, 5-ASA azo-coupled to the sulfa drug sulfapyridine) is one of colon-specific prodrugs of 5-ASA, which is in the World Health Organization’s List of Essential Medicines. Although SSZ is widely used clinically, adverse effects associated with the systemic absorption of sulfapyridine, the colon-specific carrier, can result in discontinuation and decrease the dose of SSZ. Like other 5-ASA drugs, SSZ is not effective for the treatment of severe IBD [9,10]. 

Amisulpride (ASP), an analogue of sulpiride, is an antagonist of dopamine D_2_ and D_3_ used for the treatment of schizophrenia and is thought to be pharmacologically equivalent to sulpiride [11,12]. In addition to its activity against depression and psychosis, sulpiride is a prokinetic drug that enhances GI motility without disrupting rhythm; it, thus, relieves the abdominal discomfort associated with GI disorders [13]. In addition, sulpiride may have beneficial effects on ulcers in the GI tract, including those on the tongue, stomach, and duodenum via its actions on dopamine receptors [14,15], supporting the feasible application of anti-psychotic drugs to diseases such as colitis, in which the formation of mucosal ulcers is a pathological feature [16]. In fact, we previously demonstrated that the colon-specific sulpiride glycylsulpiride alleviates rat colitis as effectively as SSZ [17].

A mutual prodrug is designed to chemically link two synergistic drugs to improve the delivery and therapeutic properties of one or both drugs [18,19]. The constituent drugs may exert synergic or additive therapeutic effects for the treatment of a target disease via distinct mechanisms of action [19]. In this study, we designed a colon-specific mutual prodrug consisting of an anti-inflammatory (anti-colitic) drug 5-ASA and ASP, with the expectation that co-delivery of the two drugs to the target site (large intestine) would elicit synergic or additive therapeutic effects on colitis. An azo bond, cleaved by microbial reductases present only in the large intestine, was introduced as the colon-specific chemical linkage between the two drugs. 5-(aminoethanoylsulfamoyl)-*N*-[(1-ethylpyrrolidin-2-yl)methyl]-2-methoxybenzamide (ASP-azo-ASA) was synthesized and tested for colon-specificity in vitro and in vivo. The anti-colitic activity of ASP-azo-ASA was compared with that of SSZ in a 2, 4-dinitrobenzenesulfonic acid hydrate (DNBS)-induced rat colitis model. To confirm that the constituent drugs elicit cooperative effects against rat colitis, anti-colitic efficacy was evaluated following rectal administration of ASP and/or 5-ASA. 

## 2. Materials and Methods 

### 2.1. Materials

ASP hydrochloride (HCl), salicylic acid, sodium nitrite (NaNO_2_), sulfamic acid, and 5-ASA were purchased from Tokyo Kasei Kogyo Co. (Tokyo, Japan). SSZ and 2, 4-dinitrobenzenesulfonic acid hydrate (DNBS) were purchased from Sigma Chemical Co. Inc. (St. Louis, MO, USA). Phosphate-buffered saline (pH 7.4, PBS) was purchased from Thermo Fisher Scientific (Waltham, MA, USA). Reaction solvents were obtained from Junsei Chemical Co. (Tokyo, Japan). All other chemicals were reagent grade, commercially available products. Spots on thin-layer chromatography (TLC) plates (silica gel F_254_s, Merck Millipore, Burlington, MA, USA) were detected using a UV lamp (at 254 nm). IR spectra were recorded using a Varian FT-IR spectrophotometer (Varian, Palo Alto, CA, USA). ^1^H-NMR spectra were recorded using a Varian AS 500 spectrometer (Varian). The chemical shifts in NMR spectra were reported in ppm downfield from tetramethylsilane.

### 2.2. Synthesis of 5-(Aminoethanoylsulfamoyl)-N-[(1-ethylpyrrolidin-2-yl)methyl]-2-methoxybenzamide (ASP-azo-ASA)

ASP HCl (2.0 mmol) was dissolved in 3.0 mL of pre-chilled 18% HCl followed by addition of NaNO_2_ (3.0 mmol), which was stirred for 1 h at 4 °C before the addition of sulfamic acid (1.0 mmol). Salicylic acid (6.0 mmol) dissolved in 0.1 M NaOH (3.0 mL) was added to the reaction system, which was then stirred at 4 °C for 2 h. The reaction was performed at room temperature for 6 h during which the pH was adjusted to 9–10. The brown powder precipitated during the reaction was filtered and washed three times with pre-chilled water and then dried in a vacuum oven. The final product was detected as one spot on TLC (acetonitrile/chloroform [5/5]). Formation of ASP-azo-ASA was verified by IR, ^1^H-NMR analysis. Yield: 85%; mp: 181–182 °C; IR (nujol mull), ν_max_ (cm^−1^): 1593 (C=O, –CONH), 1655 C=O, –COOH); ^1^H-NMR (DMSO-*d*_6_): 1.10 (t, 3*H*, *J* = 7.2 Hz), 1.22 (t, 3*H*, *J* = 7.0 Hz), 1.79 (td, 1*H*, *J*_1_ = 10.8 Hz, *J*_2_ = 6.4 Hz), 1.86 (td, 2*H*, *J*_1_ = 9.2 Hz, *J*_2_ = 4.8 Hz), 1.94 (td, 1*H*, *J*_1_ = 8.0 Hz, *J*_2_ = 4.0 Hz), 2.10 (q, 1*H*, *J* = 8.8 Hz), 2.14 (q, 1*H*, *J* = 6.8 Hz), 3.07 (m, 3*H*), 3.56 (m, 5*H*), 4.01 (s, 3*H*), 6.71 (d, 1*H*, *J* = 9.2 Hz), 7.38 (s, 1*H*), 7.74 (d, 1*H*, *J* = 9.2 Hz), 8.32 (s, 1*H*), 8.35 (s, 1*H*), 8.64 (s, 1*H*), 9.28 (s, 1*H*, broad). 

### 2.3. Apparent Distribution Coefficient and Chemical Stability

ASP-azo-ASA (100 μM) was dissolved in pH 6.8 isotonic phosphate buffer (5.0 mL, USP XXIII) pre-saturated with 1-octanol and then mixed with 1-octanol (5.0 mL, pre-saturated with pH 6.8 isotonic phosphate buffer). The solution was shaken at 200 rpm on an orbital shaker for 12 h followed by phase separation at room temperature for 4 h. The concentration of ASP-azo-ASA in the aqueous phase was determined with a UV-Vis spectrophotometer at 381 nm. The apparent distribution coefficient was calculated with the following Equation (1).

(1) $$\log\;D_{6.8}=\log(C_{OT}/C_{W})=\;\log\left[(C_{O}-C_{W}\right)/C_{W}]$$

*C_O_*: Initial concentration of the drug in isotonic phosphate buffer (pH 6.8);
*C_W_*: Equilibrium concentration of the drug in isotonic phosphate buffer (pH 6.8);
*C_OT_*: Equilibrium concentration of the drug in 1-octanol.

For the chemical stability test, ASP-azo-ASA suspended in pH 1.2 HCl buffer or in pH 6.8 isotonic phosphate buffer (100 μM, USP XXIII) was incubated at 37 °C for 10 h. A 100 μL sample of each solution was removed, and the concentrations of drugs were analyzed via high-performance liquid chromatography (HPLC). 

### 2.4. High-Performance Liquid Chromatography Analysis

The HPLC system consisting of a model 306 pump, a 151 variable UV detector, and a model 234 autoinjector from Gilson (Middleton, WI, USA) was used for experiments. A Symmetry R18 column (250 × 4.6 mm, 5 μm) and a guard column (20 × 4.6 mm, 5 μm) were purchased from Waters (Milford, MA, USA). Samples prepared for HPLC analysis were filtered through a syringe filter (0.45 μm). HPLC analysis was performed at a flow rate of 1 mL/min using a mobile phase comprising acetonitrile and 10 mM pH 4.0 phosphate buffer (1.5:8.5, *v*/*v*). The eluate was monitored at 323 nm (for 5-ASA) and 225 nm (for ASP) with the UV detector, which measured the absorption with a sensitivity of AUFS 0.01. The retention times of 5-ASA and ASP were 7.1 and 5.5 min, respectively.

### 2.5. Animals

Seven-week-old male Sprague-Dawley (SD) rats were obtained from Samtako Inc. (Gyeonggi-do, South Korea) and housed in conventional cages, acclimatized for 3–7 days under controlled temperature, humidity, and a dark/light cycle conditions. The animal protocol used in this study has been reviewed and approved by the Pusan National University–Institutional Animal Care and Use Committee (PNU-IACUC) on ethical procedures and scientific care (Approval No: PNU-2018-1898, Date 27 April 2018).

### 2.6. Incubation of ASP-azo-ASA in the Contents of the Small Intestine and Cecum

Male SD rats (250–260 g) were euthanized by CO_2_ according to the guideline for the carbon dioxide induction of the American Veterinary Medical Association (issued in 2013) and a midline incision was made to collect the contents of the proximal small intestine (PSI), distal small intestine (DSI), and cecum, separately. The intestinal contents were suspended in pH 6.8 isotonic phosphate buffer to prepare 20% (*w/v*) suspensions. The cecal contents were collected under nitrogen in a glove bag (AtmosBag, Sigma, St. Louis, MO, USA). Either ASP-azo-ASA or SSZ in pH 6.8 isotonic phosphate buffer (5.0 mL, 2.0 mM) was added to the 20% (*w/v*) suspension (5.0 mL) in conical tubes and incubated at 37 °C. Cecal contents were incubated in nitrogen. At appropriate time intervals, a 0.5 mL sample of each suspension was removed and centrifuged at 10,000× *g* at 4 °C for 5 min. Methanol (0.8 mL) was added to 0.2 mL of each supernatant, which were then vortexed and centrifuged at 20,000× *g* at 4 °C for 10 min. The concentrations of 5-ASA in the supernatants were determined by HPLC.

### 2.7. Plasma Concentration of ASP

Male SD rats were starved for 24 h with access to water. ASP (28 mg/kg) or ASP-azo-ASA (39 mg/kg) in PBS (1.0 mL) was administered by oral gavage. Blood samples were collected from the tail veins at appropriate time intervals and centrifuged at 4000 × *g* for 10 min. NaOH (1 M, 0.01 mL) was added to 0.1 mL of separated plasma, followed by the addition of ethyl acetate (0.3 mL), and centrifugation at 4000× *g* for 5 min. The organic layers were isolated, evaporated, and dissolved in the mobile phase (0.1 mL). The concentration of ASP in a 20 μL sample was determined by HPLC [20]. 

### 2.8. Induction of Colitis

Induction of colitis in rats was conducted as previously described [21,22]. Briefly, rats were starved for 24 h with free access to water, and then were lightly anesthetized with isoflurane. A rubber cannula (2.0 mm O.D.) was inserted rectally into the colon, such that the tip of the rubber cannula was placed 8 cm away from the anus, approximately at the splenic flexure. DNBS dissolved in 50% (*v*/*v*) aqueous ethanol was slowly instilled into the colon via the cannular (35 mg/0.35 mL/rat). 

### 2.9. Anti-Colitic Effects of Drugs

To evaluate the anti-colitic effects of drugs, each drug was suspended in 1.0 mL of PBS and administered to rats via oral gavage using an oral zonde (Jungdo-BNP, Seoul, South Korea). Rats were divided into four groups, each consisting of five rats, and treated as follows: Group 1 (normal group): Oral gavage of 1.0 mL PBS; group 2 (colitis group): Oral gavage of 1.0 mL PBS; group 3 (colitis + ASP-azo-ASA group): Oral gavage of ASP-azo-ASA (oral ASP-azo-ASA, 39 mg/kg, equimolar to 30 mg/kg of SSZ); group 4 (colitis + SSZ group): Oral gavage of SSZ (oral SSZ, 30 mg/kg). The same experiment was repeated independently with four groups: Group 1 (normal group): Oral gavage of 1.0 mL PBS; group 2 (colitis group): Oral gavage of 1.0 mL PBS; group 3 (colitis + ASP-azo-ASA group): Oral ASP-azo-ASA (39 mg/kg); group 4 (colitis + mixture of ASP and ASA group): Oral gavage of both ASP (28 mg/kg, equimolar to 39 mg/kg of ASP-azo-ASA) and 5-ASA (12 mg/kg, equimolar to 39 mg/kg of ASP-azo-ASA). To evaluate the anti-colitic effects of rectally administrated drugs, ASA (30 mM) and/or ASP (30 mM) suspended in pH 7.4 PBS (0.5 mL) were/was administered rectally to rats using a rubber cannula (2.0 mm O.D.). Rats were divided into five groups, each consisting of five rats and treated as follows: Group 1 (normal group): 0.5 mL of PBS; group 2 (colitis group): 0.5 mL of PBS; group 3 (colitis + ASA group): 30 mM ASA in 0.5 mL of PBS; group 4 (colitis + ASP group): 30 mM ASP in 0.5 mL PBS; group 5 (colitis + mixture of ASA and ASP group): Both 30 mM ASA and 30 mM ASP in 0.5 mM of PBS. Each drug was administered to rats once a day, 72 h after the induction of colitis. Rats were euthanized by CO_2_ after receiving treatment for 7 days. The gross colonic damage was accessed by colonic damage score (CDS) calculated by criteria previously described [21,23]. The modified scoring system was as follows: 0, normal appearance; 1, localized hyperemia but no ulcer; 2, linear ulcers without significant inflammation; 3, 2–4 cm site of inflammation and ulceration; 4, serosal adhesion to other organs, 2–4 cm site of inflammation and ulceration; 5, stricture, serosal adhesion involving several bowel loops, <4 cm site of inflammation and ulceration. CDS was assigned to each colon sample by four independent observers blinded to the drug treatment. Myeloperoxidase (MPO) activity was measured in the distal colon of rats as described previously [21]. 

### 2.10. Western Blot Analysis

To prepare tissue lysates of the inflamed distal colon, tissues (1.0 g) were disrupted and homogenized in 3.0 mL of ice-cold radioimmunoprecipitation assay buffer [50 mM Tris-HCl (pH 7.4), 1 mM EDTA, 0.7% Na deoxycholate, 1% NP-40, 150 mM NaCl, 0.3 μM aprotinin, 1 μM pepstatin, and 1 mM phenylmethylsulfonyl fluoride]. After incubation on ice for 30 min, the homogenates were centrifuged at 10,000× *g* at 4 °C for 10 min. Protein concentrations in the supernatants obtained by the above lysis process were determined using a BCA Protein Assay kit according to the manufacturer’s instructions (Pierce Biotechnology, Rockford, IL, USA). Tissue lysates were electrophoretically separated on 10% SDS-PAGE gels. Cyclooxygenase (COX)-2, and inducible nitric oxide synthase (iNOS) proteins were detected using the following antibodies: Anti-COX-2 antibody (4842S, Cell Signaling Technology, Danvers, MA, USA), anti-iNOS (NOS-2) antibody (sc-7271, Santa Cruz Biotechnology, Dallas, TX, USA). Signals were visualized using the Supersignal chemiluminescence substrate (Pierce, Rockford, IL, USA). Experiments were normalized using antibodies against α-tubulin (Santa Cruz Biotechnology, city, Dallas, TX, USA).

Western blot images were quantified using Image Lab software (version 5.2 build 14, Bio-Rad, Hercules, CA, USA). The quantification results are expressed as the mean of quantified values (*n* = 5).

### 2.11. CINC-3, TNF-α, IL-1β, and IL-6 Immunoassay

To measure levels of cytokine-induced neutrophil chemoattractant-3 (CINC-3), tumor necrosis factor-α (TNF-α), interleukin-1β (IL-1β), and interleukin-6 (IL-6) in the inflamed tissues, the inflamed distal colon was homogenized in appropriate buffers for immunoassay of each cytokine and centrifuged at 10,000× *g* at 4 °C for 10 min. An appropriate volume of the supernatant was used to determine cytokines’ levels using ELISA kits obtained from R & D Systems for CINC-3 (Minneapolis, MN, USA) and Invitrogene (Carlsbad, CA, USA). 

### 2.12. Data Analysis 

All results are expressed as the mean ± standard deviation (SD). One-way ANOVA followed by Tukey’s honestly significant difference test or the Mann–Whitney U test (for CDS) was used to test for differences between data. Differences with α or *P* < 0.05 were considered significant.

## 3. Results

### 3.1. Preparation of ASP-azo-ASA

**Figure 1:**
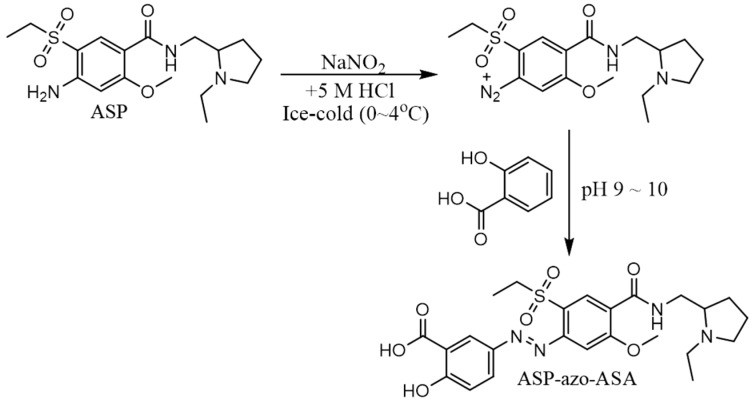
Synthesis of 5-(aminoethanoylsulfamoyl)-*N*-[(1-ethylpyrrolidin-2-yl)methyl]-2-methoxybenzamide (ASP-azo-ASA). ASP: Amisulpride, 5-ASA: 5-aminosalicylic acid.

ASP-azo-ASA was synthesized, and the reaction scheme is shown in Figure 1. The synthesis of ASP-azo-ASA was confirmed by analysis of IR and ^1^H-NMR spectra. In the IR spectra, stretching bands of the carbonyl groups were observed at 1655 cm^−1^ (–COOH in 5-ASA) and 1593 cm^−1^ (–CONH in ASP). No significant band shifts were observed for the carbonyl bands derived from the benzamide group of ASP and carboxylic group of 5-ASA. ^1^H-NMR signals originating from the aromatic protons of ASP were detected at 7.38 and 8.35 ppm, and those originating from the aromatic protons of 5-ASA were detected at 6.71, 7.74, and 8.32 ppm.

### 3.2. Oral ASP-azo-ASA Efficiently Delivers 5-ASA and ASP to the Large Intestine.

**Figure 2:**
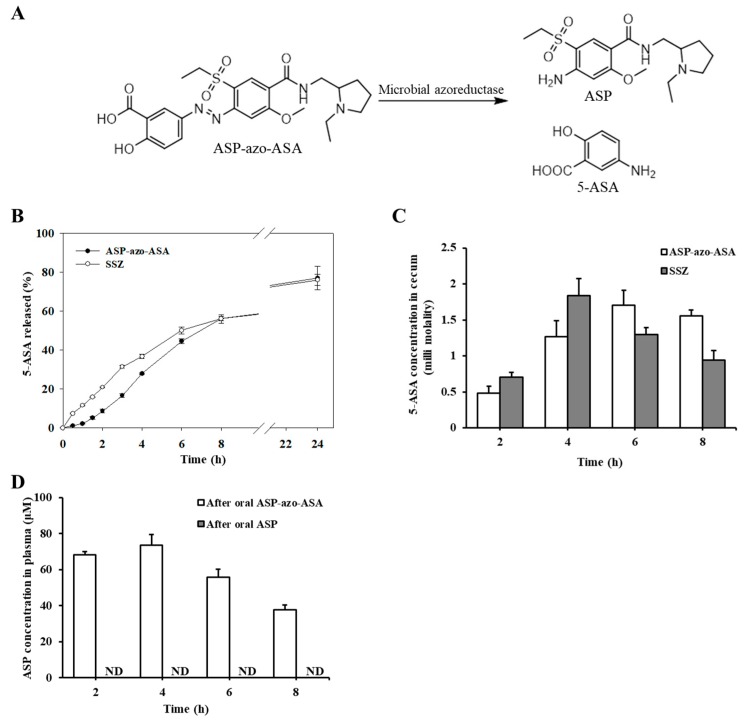
ASP-azo-ASA is a colon-specific prodrug activated to form ASP and 5-ASA. (**A**) Activation of ASP-azo-ASA by microbial azoreductase in large intestine. (**B**) ASP-azo-ASA (1 mM) or sulfasalazine (SSZ, 1 mM) was incubated in cecal contents and a mixture of small intestinal contents was suspended in pH 6.8 isotonic phosphate buffer (10%). At appropriate time intervals, the levels of 5-ASA in the samples were determined by high-performance liquid chromatography (HPLC). Data represent the percentage of 5-ASA released from ASP-azo-ASA or SSZ. (**C**) Male Sprague-Dawley rats (250–260 g) were starved for 24 h and allowed access to water. SSZ (30 mg/kg) or ASP-azo-ASA (39 mg/kg, equimolar to 30 mg of SSZ) suspended in PBS (1.0 mL) was administered to rats by oral gavage. The rats were sacrificed, and a midline incision was made to obtain the cecal contents. Drugs in the cecal contents were analyzed by HPLC. (**D**) ASP (28 mg/kg, equimolar to 39 mg of ASP-azo-ASA) or ASP-azo-ASA (39 mg/kg) suspended in PBS (1.0 mL) was administered orally to rats and blood was collected at predetermined intervals from the tail vein. Blood ASP levels were analyzed by HPLC. ND: Not detectable. The data in (**B**), (**C**) and (**D**) represent the mean ± standard deviation (*n* = 5).

We examined the colon-specificity of ASP-azo-ASA. Its distribution coefficient was −0.6376 in a 1-octanol/pH 6.8 buffer system, indicating that ASP-azo-ASA is hydrophilic and that its passive diffusion through the epithelial layer of the GI tract is not efficient. When ASP-azo-ASA was incubated in the contents of the small intestinal and in buffers of pH 1.2 and 6.8, ASP was not detected and there was no change in the concentration of ASP-azo-ASA. In contrast, ASP-azo-ASA was cleaved to form ASP and 5-ASA in the cecal contents (the scheme for the cecal activation of ASP-azo-ASA is shown in Figure 2A), and the percentage of ASP-azo-ASA cleavage was approximately 27% at 4 h and 56% at 8 h (Figure 2B). The conversion rate of ASP-azo-ASA to 5-ASA was compared with that of SSZ. As shown in Figure 2B, the rates of ASP-azo-ASA and SSZ conversion were comparable, and the percent conversion of the two prodrugs was similar at 24 h, although SSZ was converted faster in the early phase. The cecal conversion of ASP-azo-ASA to ASP is shown in Figure S1.

To further examine the colon-specificity of ASP-azo-ASA, 5-ASA was measured in the cecum at predetermined time intervals following oral ASP-azo-ASA, which was repeated with oral SSZ for comparison. As shown in Figure 2C, 5-ASA was detected in the cecum at 2 h following oral SSZ and oral ASP-azo-ASA. Peak concentrations of 5-ASA were observed 4 h after oral SSZ, and at 6 h after oral ASP-azo-ASA. Overall, the amount of 5-ASA delivered to the cecum was similar.

In general, colonic delivery of a drug reduces its systemic absorption, reducing the potential of the drug to elicit systemic side effects [7]. To examine this, the concentration of ASP in blood was monitored following oral ASP-azo-ASA, and the same experiment was repeated with oral gavage of ASP (oral ASP). As shown in Figure 2D, although a substantial amount of ASP accumulated in the cecum following oral administration of ASP-azo-ASA, ASP was barely detected in the blood. In contrast, up to about 73 μM ASP was detected following oral ASP. 

### 3.3. ASP-azo-ASA Is More Effective Than SSZ at Ameliorating Rat Colitis

**Figure 3:**
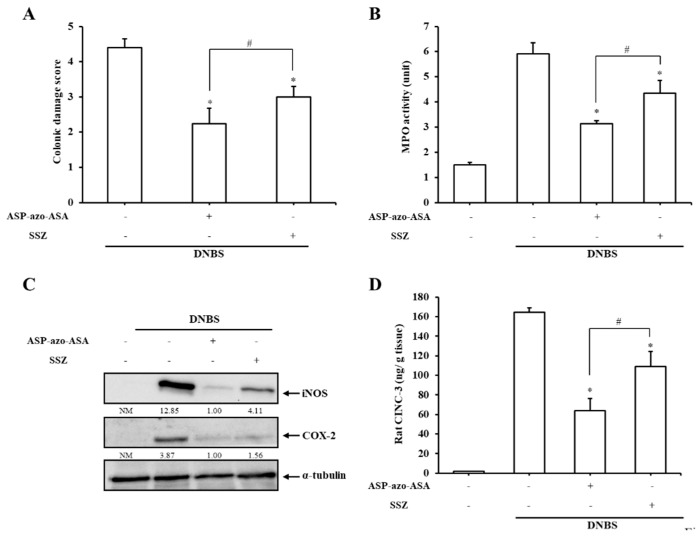
ASP-azo-ASA is more effective than SSZ at ameliorating rat colitis. Three days after the induction of colitis by 2, 4-dinitrobenzenesulfonic acid hydrate (DNBS), ASP-azo-ASA (39 mg/kg), or SSZ (30 mg/kg) suspended in PBS (1.0 mL) was administered to rats by oral gavage once per day and the rats were sacrificed after 7 days of treatment. (**A**) Colonic damage score (CDS) was determined. *: α < 0.05 vs. the TNBS control (**B**). Myeloperoxidase (MPO) activity was measured using the inflamed distal colon (4 cm). The levels of the inflammatory mediators iNOS, COX-2 (**C**), and CINC-3 (**D**), were assessed in the inflamed colon. *P < 0.05 vs. control, NM: Not measurable. The data in (**A**), (**B**), and (**D**) represent the mean ± standard deviation (*n* = 5). * *P* < 0.05 vs. control, # *P* < 0.05.

We investigated whether ASP-azo-ASA could act as a mutual prodrug against colitis. To examine this, ASP-azo-ASA (39 mg/kg, equimolar to 30 mg/kg SSZ) was administered via oral gavage once a day, three days after the induction of colitis by DNBS. The experiment was repeated with oral SSZ (30 mg/kg), which is composed of 5-ASA and sulfapyridine and exerts anti-colitic effects that are solely dependent on 5-ASA. CDS and MPO activities were assessed seven days after oral gavage of the prodrugs. As shown in Figure S2 (representative images of the distal colons) and Figure 3A (CDS), the distal colons of animals from the DNBS control group (with no medication) were severely damaged, accompanied by hemorrhagic mucosal destruction, intraluminal stricture, edema, and extensive serosal adhesion to other organs. ASP-azo-ASA markedly prevented the colonic damage, as assessed by CDS. In parallel, as shown in Figure 3B, ASP-azo-ASA decreased MPO activity in the inflamed distal colon up to about 47% of the DNBS control group. ASP-azo-ASA was more effective than SSZ at improving CDS and MPO activities. To further examine the anti-colitic superiority of ASP-azo-ASA to SSZ, levels of inflammatory mediators were determined and compared in the inflamed distal colon following oral ASP-azo-ASA and oral SSZ. As shown in Figure 3C,D, consistent with the results for CDS and MPO activities, ASP-azo-ASA decreased the levels of the inflammatory mediators iNOS, COX-2, and CINC-3 more effectively than SSZ. Levels of inflammatory cytokines, interleukin-6 (IL-6), tumor necrosis factor-α (TNF-α), and IL-1β in the inflamed distal colon were measured and a significant decrease in the cytokines was observed, where ASP-azo-ASA was more effective than SSZ, as well (Figure S3A–C). To examine whether the anti-colitic effects of ASP-azo-ASA were associated with the colonic delivery of 5-ASA and ASP, the above experiments performed to assess anti-colitic activity were repeated using a mixture of 5-ASA and ASP. Anti-colitic activity was assessed following oral gavage of either ASP-azo-ASA (39 mg/kg) or a mixture of 5-ASA (12 mg/kg) and ASP (28 mg/kg), equimolar to ASP-azo-ASA (39 mg/kg). As shown in Figure S4A (representative images of distal colons), B (CDS), C (MPO), D (COX-2 and iNOS), and E (CINC-3), no significant anti-colitic effects were observed upon oral gavage of the 5-ASA and ASP mixture in contrast to those observed upon oral ASP-azo-ASA.

### 3.4. Combined Intracolonic Treatment with 5-ASA and ASP Alleviates Rat Colitis in an Additive Manner

**Figure 4:**
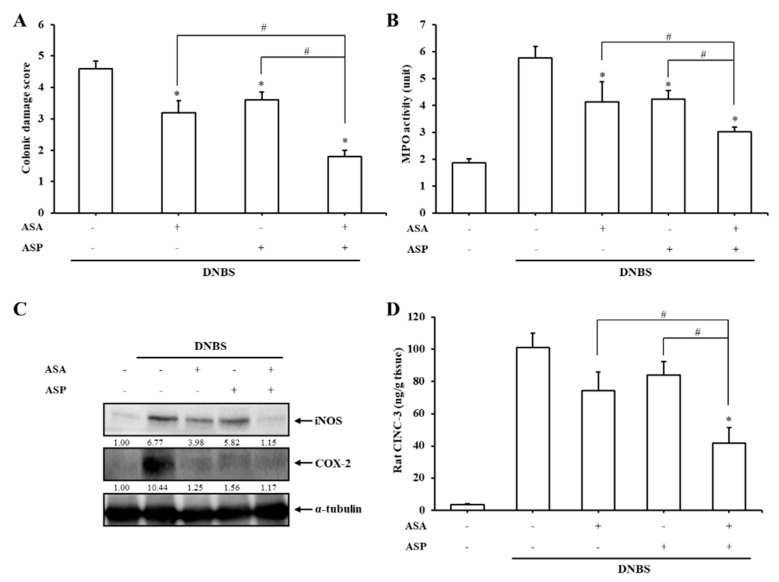
Combined intracolonic treatment with 5-ASA and ASP alleviates rat colitis in an additive manner. Three days after the induction of colitis by DNBS, 5-ASA (30 mM) and/or ASP (30 mM) dissolved in PBS (0.5 mL) were/was administered rectally to rats once per day and the rats were sacrificed after 7 days of treatment. (**A**) CDSs were determined. *: α < 0.05 vs. the TNBS control (**B**). MPO activity was measured using the inflamed distal colon (4 cm). The levels of inflammatory mediators, iNOS, COX-2 (**C**), and CINC-3 (**D**), were assessed in the inflamed colon. * *P* < 0.05 vs. control. The data in (**A**), (**B**), and (**D**) represent the mean ± standard deviation (*n* = 5). * *P* < 0.05 vs. control, ^#^ *P* < 0.05.

Our data showed that oral ASP-azo-ASA was more effective than oral SSZ at ameliorating colitis, suggesting that ASP-azo-ASA acts as a mutual prodrug. To verify the mutual activity of 5-ASA and ASP against rat colitis, ASP and/or 5-ASA were/was administered rectally once a day for seven days mimicking the therapeutic situation in which oral ASP-azo-ASA exerted anti-colitic effects in colitic rats [24]. As shown in Figure S5 (representative images of distal colons), Figure 4A (CDS), Figure 4B (MPO), Figure 4C (iNOS and COX-2), and Figure 4D (CINC-3), combined intracolonic treatment with 5-ASA and ASP was significantly more effective at improving all inflammatory indices studied (except for COX-2) in the inflamed colons compared to treatment with ASP or 5-ASA individually. 

## 4. Discussion

In this study, a colon-specific mutual prodrug was designed, which coupled 5-ASA to ASP via an azo-bond, a typical chemical linkage for colon-specific activation, in order to improve the anti-colitic activity of 5-ASA [7]. ASP was chosen as a partner for the mutual prodrug because sulpiride, which is pharmacologically equivalent to ASP, presents anti-gastric ulcer and anti-colitic activities [14,17] and ASP possesses an aniline moiety suitable for azo-coupling with salicylic acid to produce a colon-specific prodrug that liberates ASP and 5-ASA. In fact, ASP-azo-ASA was readily synthesized and activated to ASP and 5-ASA in the large intestine. Moreover, ASP-azo-ASA transversed the upper GI tract and reached the large intestine as efficiently as SSZ, as demonstrated by the observation that oral ASP-azo-ASA delivers an amount of 5-ASA to the cecum, which is comparable to that delivered by oral SSZ. Consistent with a general feature of drugs delivered specifically to large intestine via the oral route [7,17,21], ASP was barely detectable in the blood when administered as oral ASP-azo-ASA, although 73 μM ASP was detected in the blood following oral ASP at an equimolar dose. This suggests that ASP-azo-ASA may be used for the treatment of colitis with potentially fewer systemic (side) effects than ASP, which is an important consideration for the therapeutic switching of a drug [17,25]. 

Due to SSZ, 5-ASA azo-linked to sulfapyridine, being a colon-specific prodrug composed of the therapeutically active 5-ASA and therapeutically inert sulfapyridine against colitis [26,27], our data showed that oral ASP-azo-ASA was more effective than oral SSZ at ameliorating colonic damage and inflammation suggesting that ASP-azo-ASA acts as a mutual prodrug against colitis. This was verified by our finding that rectal administration of 5-ASA plus ASP was therapeutically superior to treatment with 5-ASA or ASP alone. 

Consistent with the general advantages of colon-specific delivery for the treatment of colonic diseases, such as IBD [7,8], oral ASP-azo-ASA was effective against colitis while oral gavage of a mixture of ASP and 5-ASA did not elicit significant anti-colitic effects. Colon-specific drug delivery provides a greater concentration of drug at the target site than does conventional drug delivery when the same dose of the drug is administered via the oral route. In addition, the duration of drug action at the target site can be increased with colon-specific delivery, considering the transit time of the large intestine [7,8]. These pharmacokinetic features may explain the therapeutic difference between orally administered ASP-azo-ASA and the physical mixture. In favor of this argument, rectal administration of ASP and 5-ASA, which mimics the therapeutic situation of oral ASP-azo-ASA [7,24], was also effective against colitis.

ASP presents anti-colitic activity, although it requires rectal delivery. This suggests that the anti-colitic activity of ASP may be independent of its anti-psychotic pharmacology and antagonistic activity on dopamine receptors. The dose of ASP used for oral gavage was higher than that usually used for anti-psychosis treatment [11]. Nevertheless, the pharmacological mechanism(s) underlying the anti-colitic activity of ASP may get activated when it is present at higher concentrations, which can be obtained by colon-specific delivery or rectal administration, but not by conventional delivery. The duration of ASP action at the target site may also explain the therapeutic difference between rectal and oral ASP administration. Further studies are required to reveal the pharmacological mechanism(s) underlying the anti-colitic effects of ASP and the therapeutic alliance of ASP and 5-ASA against colitis.

## 5. Conclusions

ASP-azo-ASA may be a colon-specific mutual prodrug resulting in enhanced anti-colitic activity of 5-ASA for the treatment of colitis.

## Supplementary Materials

The following are available online at https://www.mdpi.com/1999-4923/11/11/585/s1, Figure S1: ASP-azo-ASA is a colon-specific prodrug activated to form ASP and 5-ASA, Figure S2: Representative images of the distal colons of rats following oral gavage of drugs. Figure S3: ASP-azo-ASA is more effective than SSZ in decreasing the levels of inflammatory cytokines in the inflamed distal colon of rats Figure S4: Anti-colitic effects of ASP-azo-ASA are associated with colonic delivery of ASP and 5-ASA. Figure S5: Representative images of the distal colons of rats following rectal administration of drugs.
