# Supplementary material for: Conjugation of Amisulpride, an Anti-Psychotic Agent, with 5-Aminosalicylic Acid via an Azo Bond Yields an Orally Active Mutual Prodrug against Rat Colitis
Source: Pharmaceutics. 2019 Nov 7;11(11):585. doi: 10.3390/pharmaceutics11110585 (PMC6920822; doi:10.3390/pharmaceutics11110585)
Supplement: Supplementary file 1 [file pharmaceutics-11-00585-s001.pdf]

pharmaceutics

Supplementary Materials: Conjugation of Amisulpride, an Anti-Psychotic Agent, with 5-Aminosalicylic Acid via an Azo Bond Yields an Orally Active Mutual Prodrug against Rat Colitis

Wooseong Ki, Dayoon Kim, Seongkeun Jeong, Sanghyun Ju, Hanju Lee, Soojin Kim, Jin-Wook Yoo, In-Soo Yoon and Yunjin Jung

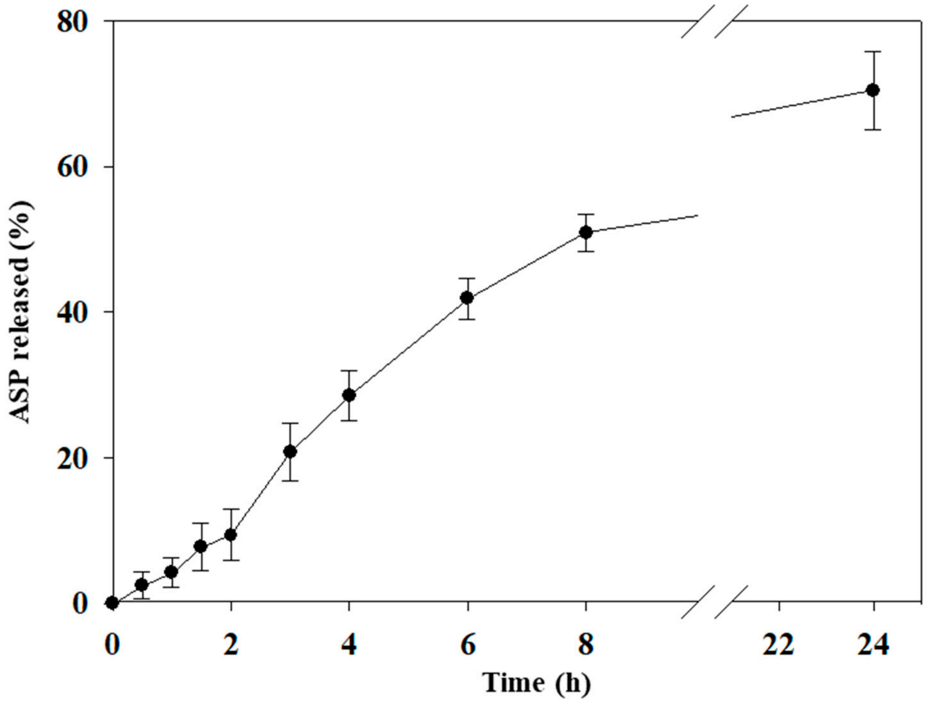

Figure S1. ASP-azo-ASA is a colon-specific prodrug activated to form ASP and 5-ASA.

ASP-azo-ASA (1.0 mM) was incubated in cecal contents suspended in pH 6.8 isotonic phosphate buffer (10%). At appropriate time intervals, the levels of ASP in the samples were determined by high-performance liquid chromatography. Data represent the percentage of ASP released from ASP-azo-ASA and are represented as the mean ± standard deviation (n = 5).

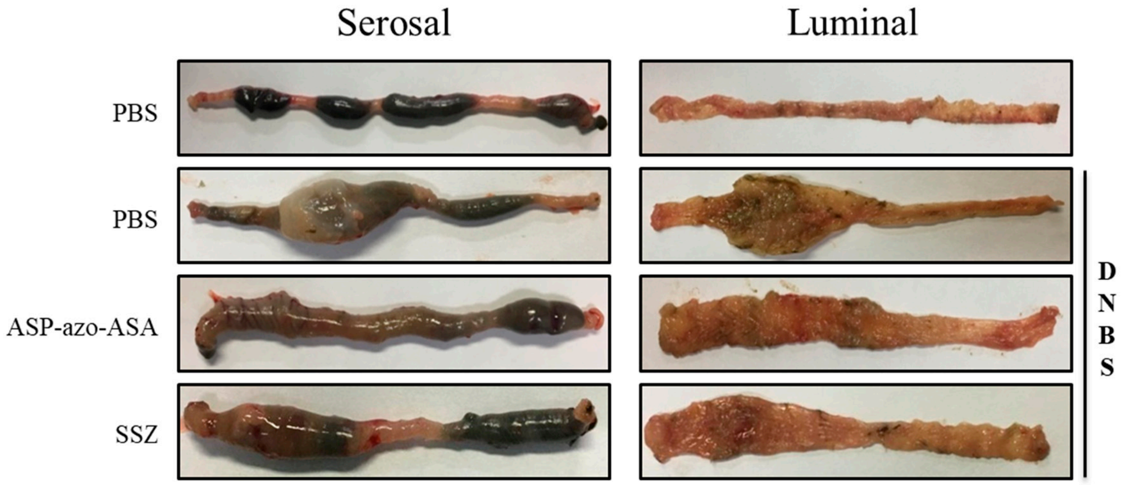

Figure S2. Representative images of the distal colons of rats following oral gavage of drugs.

Three-days after the induction of colitis by DNBS, ASP-azo-ASA (39 mg/kg) or SSZ (30 mg/kg) suspended in PBS (1.0 mL) was administered to rats by oral gavage once per day and the rats were sacrificed after 7 days of treatment. The serosal and luminal sides of isolated distal colons were photographed.

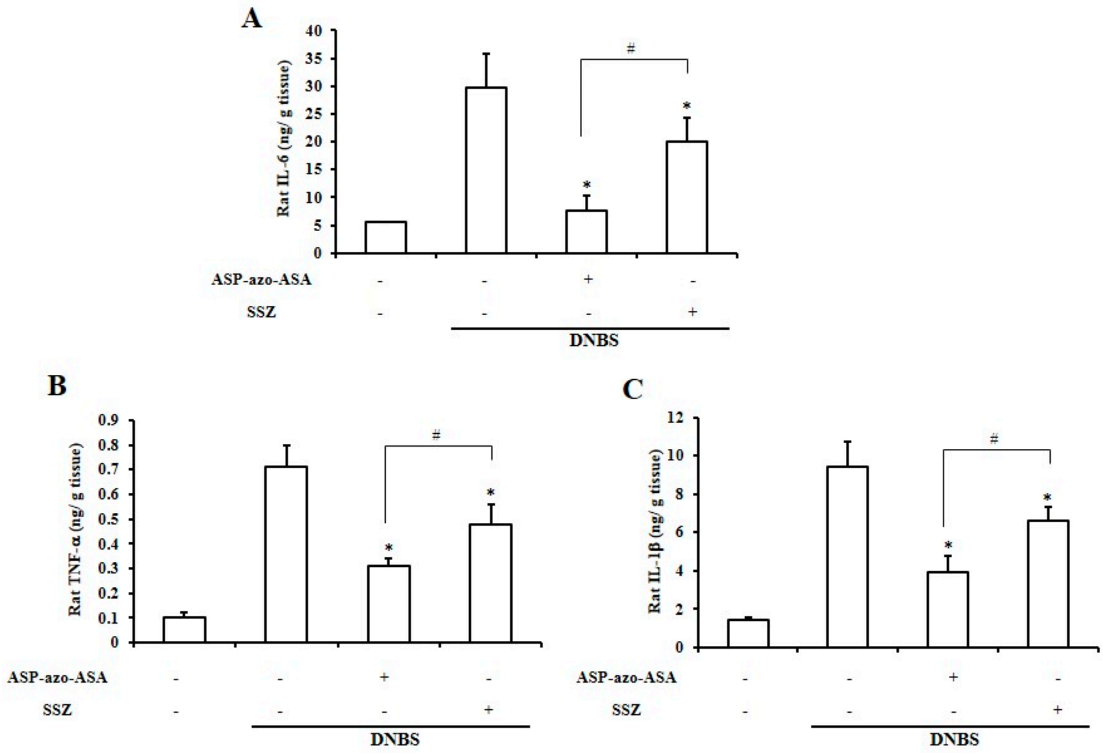

Figure S3. ASP-azo-ASA is more effective than SSZ in decreasing the levels of inflammatory cytokines in the inflamed distal colon of rats. To measure levels of interleukin-6 (**A**, IL-6), tumor necrosis factor-α (**B**, TNF-α), and IL-1β (**C**) in the inflamed tissues, the inflamed distal colon was homogenized in appropriate buffers for immunoassay of each cytokine and centrifuged at 10,000 ×*g* at 4 °C for 10 min. An appropriate volume of the supernatant was used to determine cytokines' levels using ELISA kits. The data represent the mean ± standard deviation ($n = 5$). * $P < 0.05$ vs control, # $P < 0.05$.

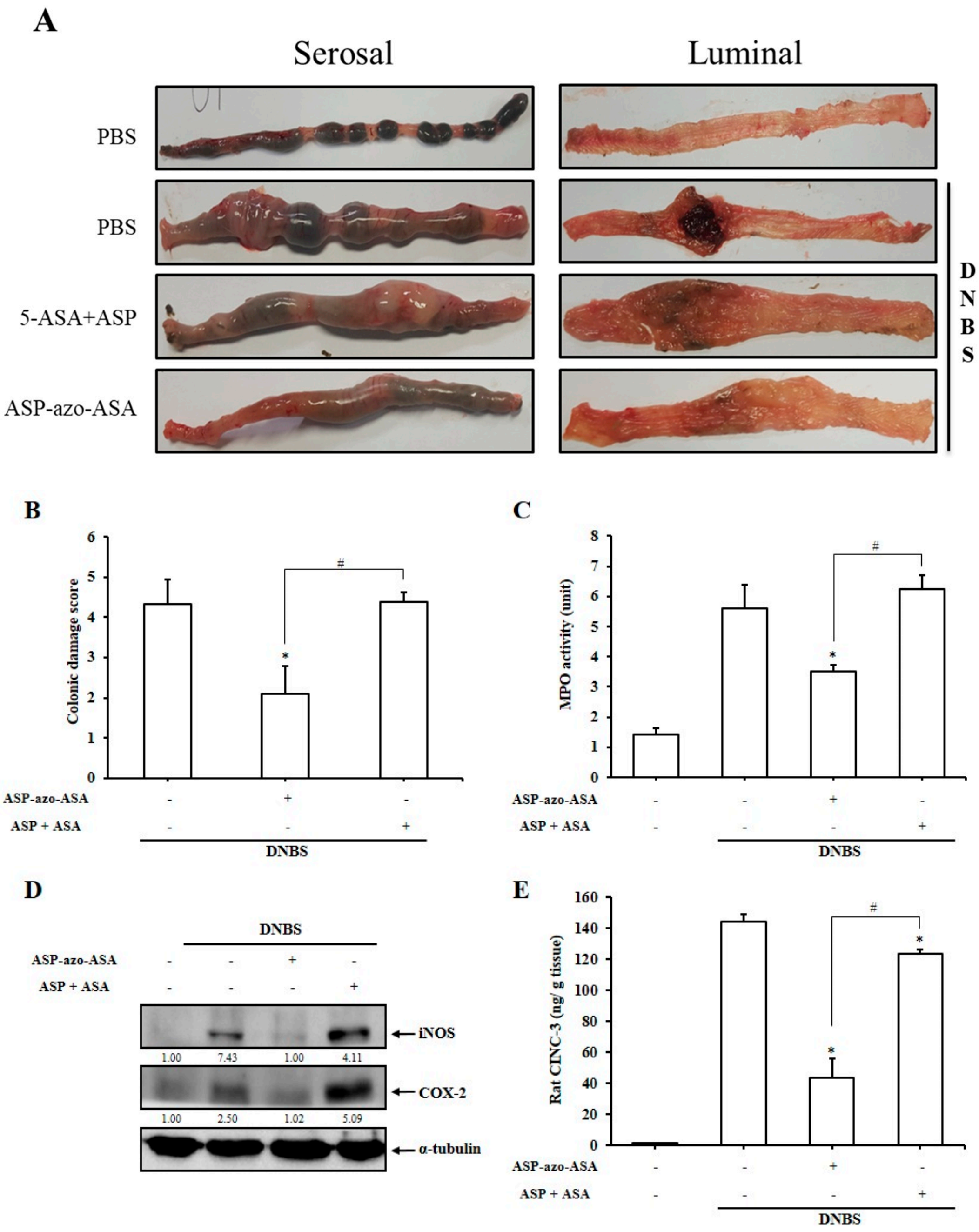

Figure S4. Anti-colitic effects of ASP-azo-ASA are associated with colonic delivery of ASP and 5-ASA. Three-days after the induction of colitis by DNBS, ASP-azo-ASA (39 mg/kg) or a mixture of 5-ASA (12 mg/kg) and ASP (28 mg/kg) suspended in PBS (1.0 mL) was administered to rats by oral gavage once per day and the rats were sacrificed after 7 days of treatment. (**A**) Representative images of the serosal and luminal sides of isolated distal colons (**B**). The CDS was determined. *: $\alpha < 0.05$ vs. the DNBS control (**C**). MPO activity was measured using inflamed distal colons (4 cm). The levels of inflammatory mediators, iNOS, COX-2 (**D**), and CINC-3 (**E**), were assessed in the inflamed colon. * $P < 0.05$ vs control. The data in **B**, **C**, and **E** represent the mean ± standard deviation ($n = 5$). * $P < 0.05$ vs control, # $P < 0.05$.

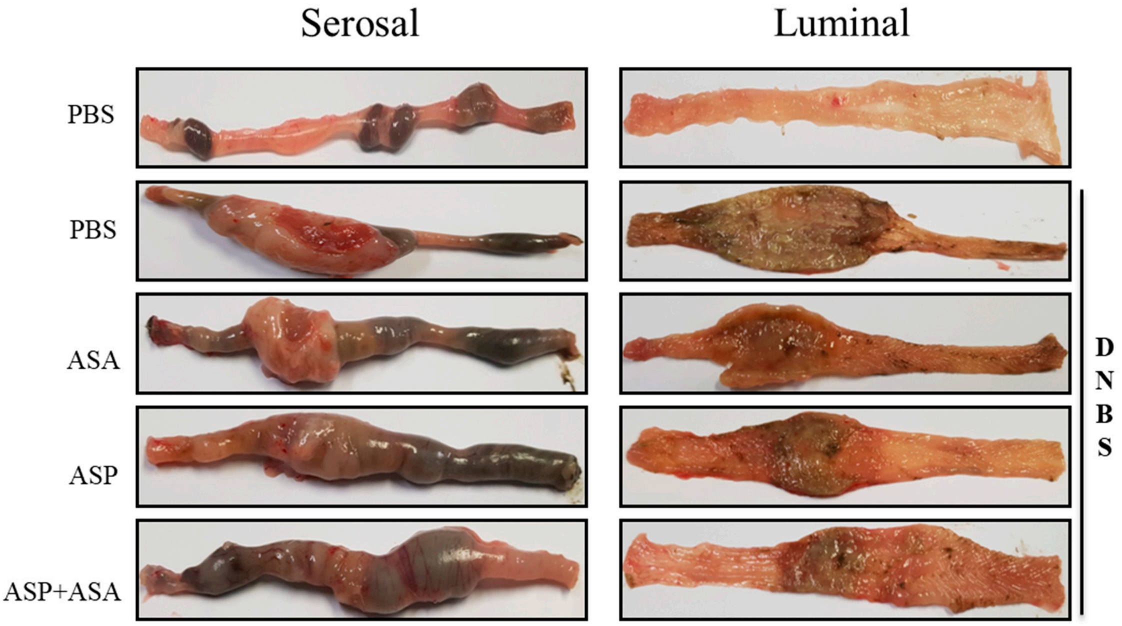

Figure S5. Representative images of the distal colons of rats following rectal administration of drugs.

Three-days after the induction of colitis by DNBS, 5-ASA (30 mM) and/or ASP (30 mM) suspended in PBS (0.5 mL) at pH 7.4 were/was administered rectally to rats once per day and the rats were sacrificed after 7 days of treatment. The serosal and luminal sides of isolated distal colons were photographed

© 2019 by the authors. Submitted for possible open access publication under the terms and conditions of the Creative Commons Attribution (CC BY) license (http://creativecommons.org/licenses/by/4.0/).
